# Rethinking scholarship in medical education during the era of the COVID-19 pandemic

**DOI:** 10.15694/mep.2020.000097.1

**Published:** 2020-05-13

**Authors:** Poh-Sun Goh, John Sandars

**Affiliations:** 1 National University of Singapore; 2 Edge Hill University Medical School

**Keywords:** Scholarship in medical education, action research, educational design research, transformational change, COVID-19

## Abstract

This article was migrated. The article was marked as recommended.

The COVID-19 pandemic has significantly disrupted society and communities across the world requiring new and innovative approaches for healthcare, work, education and leisure. Similar changes have been precipitated in medical education, producing a rapid and major impact on students, educators and institutions. However, institutions still require educators to engage with scholarship in medical education, including providing evidence for promotion and tenure. We propose that resolving this tension between the demands of delivering a high quality curriculum and maintaining scholarship in medical education during the era of the COVID-19 pandemic requires urgent consideration of a transformational change in the scholarship in medical education. Key aspects of this change are a focus on rapid cycles of research to inform teaching, with local and wider dissemination using newer rapid approaches to publication and social media, and acceptability of these changes by institutions.

## Introduction

The COVID-19 pandemic has triggered an immense and extraordinary change to society and communities across the world requiring new and innovative approaches for healthcare, work, education and leisure. A major aspect of this change has been the rapid, and potentially permanent, shift in the use of technology to ensure that healthcare, work, education and leisure activities can be maintained.

Medical education has been no exception to these changes, with a similar rapid shift in the use of technology for delivery of all aspects of the curriculum, from teaching to virtual clinical training to assessments, and across the continuum of medical education. Educators, learners and institutions have had to adjust to these changes and manage the various increased demands related to workload, new practices and external regulatory authorities. However, at the same time, educators are expected to continue their active engagement with scholarship in medical education, including providing evidence for promotion and tenure.

In this Personal View, we propose that a transformational change in how we consider scholarship in medical education during the era of the COVID-19 pandemic is urgently required. We consider that this change can help to reduce the tension between the demands of rapidly responding to the demands of maintaining the delivery of a high quality curriculum and the need to continue with scholarship in medical education.

## Scholarship in medical education

Our understanding of scholarship in medical education has been informed by several authors, with each providing different key components. Boyer (1990) described four categories of scholarship: discovery and creation, integration, application and teaching. Several criteria of how scholarship can be assessed were proposed by Glassick (2000), including clear goals, adequate preparation, appropriate methods, significant results, clear presentation, and reflective critique. Additional features were presented by Hutchings and Shulman (1999), including wider dissemination in a form suitable for review and evaluation, which is accessible for exchange with other members of education community. Institutions have widely adopted these different components of scholarship and there is an expectation that medical educators engage in the entire process and provide appropriate evidence of this process.

**Figure 1.**
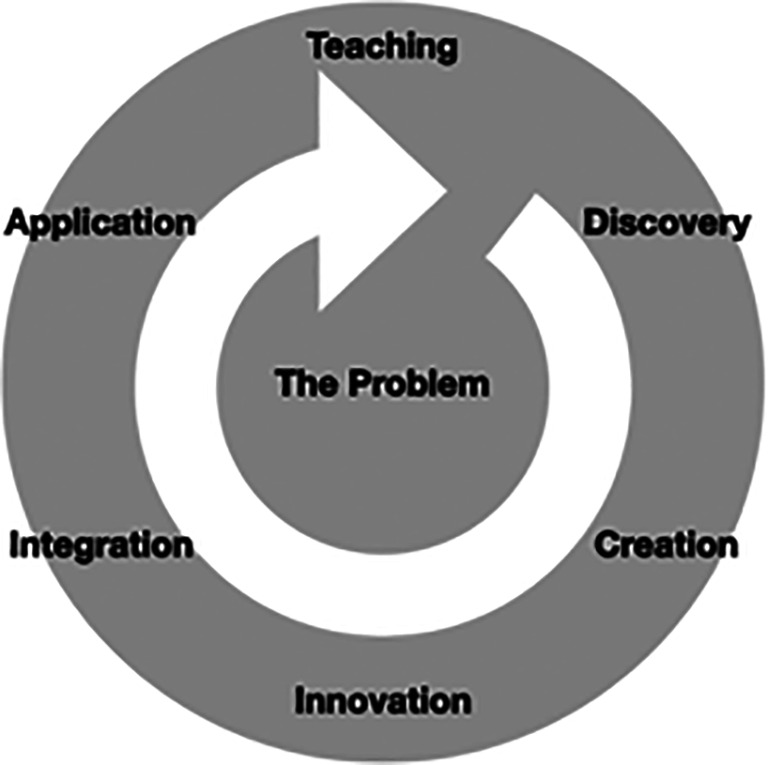
The iterative scholarship cycle

Scholarship in medical education is often an iterative cycle, with the experience of teaching identifying problems which leads to a research phase of discovery for the creation of new knowledge and understanding about the problem. These findings can lead to the innovation of new approaches for teaching through the process of integration with a variety of sources of knowledge, including colleagues and a review of the literature. Following application of the new approach to teaching, the cycle can be repeated (see Figure 1). Throughout the iterative cycle, there are opportunities to evidence scholarship through scholarly outputs, such as producing and disseminating an article that describes the intervention and findings.

## The challenge of scholarship in the era of the COVID-19 pandemic

The rapidity of the evolving disruption and response evoked by the COVID-19 pandemic on medical education requires educators, and also institutions, to constantly monitor and make changes to ensure that the delivery of the curriculum is appropriate and achieving its vision, aims and intended outcomes. The process of monitoring progress identifies problems that are key to initiating the scholarship cycle. However, over time, these problems are likely to move from being simple to complex. Alternative methods for research, such as action research and educational design research, which use rapid cycles of investigation and implementation, will be required to answer these problems, but these methods are less familiar to institutions and also to the editors and peer reviewers of many journals. In addition, the dissemination required for evidence of scholarship through publications in high impact journals can be a lengthy process, even in those journals with early pre-publication, and these journals also usually do not have open access.

### Research for scholarship

The COVID-19 pandemic has required increased use of online methods to deliver the curriculum and Satya Nadella, Microsoft’s Chief Executive Officer, has observed “two years worth of digital transformation in two months” ( Spataro, 2020). A curriculum can be considered as a syllabus of content and the variety of methods to achieve predefined learning outcomes. However, a curriculum is also an expression of the core values of the institution, such as equity for the learners and social accountability.

Several categories of problems are recognised but an important distinction can be made between simple and complex ( Snowdon, 1999). Simple problems identified through online delivery of the curriculum will have simple research questions, such as what is the impact of student learning outcomes by the use of videos created from PowerPoint presentations. A single iterative research cycle can help to answer the question but also inform further development of the use of videos. However, complex problems identified through online delivery will be more difficult to research, such as how can equity for learners be maintained when there are multiple -inter-related factors contributing to equity (such as gender, socio-economic circumstances and cultural background). Effective online delivery of the curriculum also has multiple inter-related factors that have to be aligned in the design and implementation phase of the intervention, including the learner, the educator, the intended educational outcomes, the content, the available technology, the instructional design and the organisational context ( Sandars *et.al.*, 2020). For complex problems, iterative research methods over several cycles are essential to increasingly modify the educational intervention to ensure that it is effective ( Hanington and Martin, 2019). An essential aspect of this iterative approach is the use of design thinking to produce and innovate creative potential solutions ( Badwan *et al*., 2018). Because of the rapidly evolving impact of the COVID-19 pandemic on medical education, these iterative cycles also have to rapidly conducted.

### Dissemination for scholarship

Dissemination of scholarly outputs, which are essential for the demonstration of scholarship in medical education, has relied mainly on journals and conferences but the frequency and time required for publication has become increasingly inappropriate to inform the wider community of medical educators who need timely new knowledge to inform their responsibilities for delivery of the curriculum. In addition, many journals and conference presentations are not open access and this limits the reach of global dissemination, especially for low and middle income countries.

## Responding to the challenge of scholarship in the era of the COVID-19 pandemic

Our recommendations for responding to the challenge can be considered within three major themes: research for scholarship, dissemination for scholarship and the institution.

### Research for scholarship

The highly iterative nature of action research and educational design research will likely add to, and accelerate, the scholarship of discovery and creation ( Coghlan and Brannick, 2019; McKenney and Reeves, 2018). Many of the criticisms about action research and educational design research, especially about their appropriateness for scholarship, are about the rigor of the data collection and analysis but this can be easily rectified by careful attention to detail, such as following standard guidance on performing qualitative and quantitative research.

**Figure 2.**
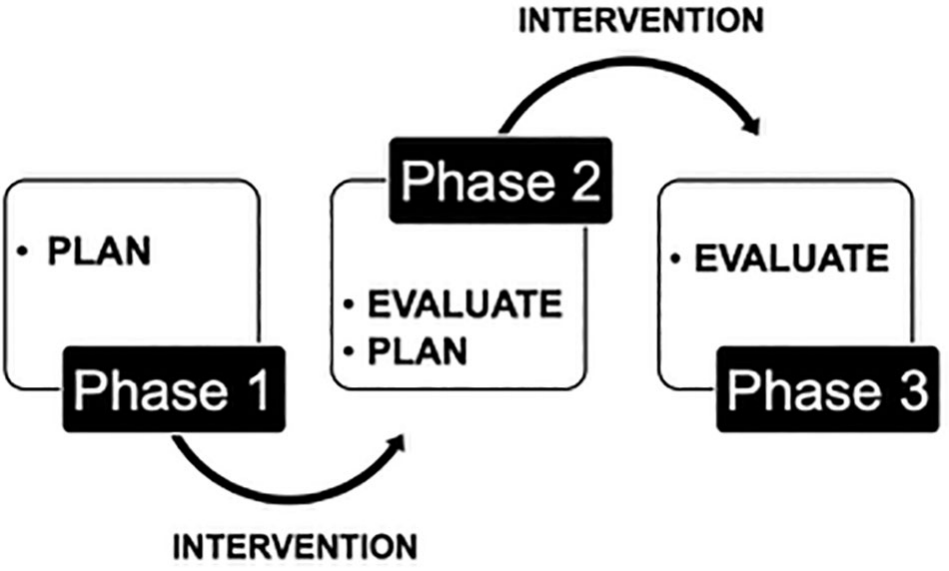
The iterative research cycle

Action research and educational design research have the intention to simultaneously implement an educational intervention (action) and to understand the various factors that contribute to the educational impact of the intervention (research). Through iterative cycles, the intervention is evaluated and modified to achieve its intended impact and each cycle of planning, implementation and evaluation informs the next cycle (see Figure 2).

The research component, which is the creation of new knowledge and understanding though the evaluations, can directly inform the development of teaching but also the findings can be more widely disseminated to inform the work of other medical educators.

### Dissemination for scholarship

Providing evidence of scholarship through scholarly outputs in medical education is a high priority for most institutions and subsequently for most educators. The use of technology to support educational scholarship has been called digital scholarship ( Goh and Sandars, 2019). There are several online approaches for rapid publishing and dissemination, especially open access publication and social media. Opportunities for rapid open access publishing has become increasingly available, with some offering pre-publication peer review and others offering post-publication peer-review. Similarly, there has been increasing opportunities using a variety of social media, such as Facebook and Twitter. There are well established metrics to inform digital scholarship and educators can create portfolios to curate these metrics as evidence ( Cabrera *et al*., 2017).

An important aspect of the dissemination of scholarly works is to ensure that it supports decision-making about their teaching and delivery of the curriculum, such as ensuring that scholarly outputs will focus on examining what works and what does not work from their experience of the interventions. More explicit reporting of this information, including helping and hindering factors, can be facilitated by the use of templates, such as SQUIRE-EDU (Standards for QUality Improvement Reporting Excellence in Education) ( Ogrinc *et al*., 2019) or TIDieR (Template for Intervention Description and Replication) ( Cotterill *et. al.*, 2019).

### The institution

There are important challenges for institutions in the implementation of our proposed transformational change for scholarship in medical education. The main challenges are likely to be greater acceptance of the action research or design research paradigm and also recognition of the evidence and metrics of scholarship which are produced from dissemination in open access publications and social media. Acceptance of these challenges is essential for taking full advantage of the rapid cycle iterative investigation and evaluation cycles that underpin the action research and design research paradigm. This evolution will need training and time to fully adopt, for senior faculty and academic administrators.

Educators will also need to have sufficient time to continue their scholarly activities. In an environment with multiple demands, including healthcare, teaching, academic and family, it is essential that opportunities for research and dissemination are both relevant to the urgent need to deliver the curriculum but can also satisfy the demands of the institution to evidence scholarship in medical education. An important opportunity of combining teaching with research are learning communities of educators, with demonstrable impact on the academic achievement of learners ( Vescio *et al.*, 2008).

## Conclusion

The COVID-19 pandemic has significantly disrupted medical education, producing a rapid and major impact on students, educators and institutions. However, institutions still require educators to engage with scholarship in medical education, including providing evidence for promotion and tenure. We propose that resolving this tension between the demands of delivering a high quality curriculum and maintaining scholarship in medical education during the era of the COVID-19 pandemic requires urgent consideration of a transformational change in the scholarship in medical education. Key aspects of this change are a focus on rapid cycles of research to inform teaching, with local and wider dissemination using newer rapid approaches to publication and social media. There are important challenges for institutions in the implementation of our proposed transformational change. Resolving these challenges has important implications for both the scholarship of medical education during the COVID-19 pandemic but also there are likely to be long term permanent changes in how both educators and their institutions conceptualise and engage in scholarship.

## Take Home Messages

• The COVID-19 pandemic has produced rapid and evolving changes in the delivery of the curriculum
• We propose that scholarship in medical education, with the need to engage with research and produce evidence, requires a transformational change
• Rapid iterative cycles of research by action research and education design research are more appropriate
• Dissemination of scholarly work in rapidly changing environments requires fast and open access publication
• There are important challenges for institutions in the implementation of our proposed transformational change

## Notes On Contributors

**Poh Sun Goh**, MBBS, FRCR, FAMS, MHPE, FAMEE, is an Associate Professor and Senior Consultant Radiologist at the Yong Loo Lin School of Medicine, National University of Singapore, and National University Hospital, Singapore. He is a graduate of the Maastricht MHPE program, a member of the AMEE TEL committee, and a Fellow of AMEE. ORCiD: https://orcid.org/0000-0002-1531-2053

**John Sandars** MB ChB (Hons), MSc, MD, MRCP, MRCGP, FAcadMEd, CertEd, FHEA is Professor of Medical Education and Director of Medical Education Innovation and Scholarship at Edge Hill University Medical School, Ormskirk, UK, and is Co-Chair of the AMEE Technology Enhanced Learning Committee. ORCiD: https://orcid.org/0000-0003-3930-387X
