# Supplementary material for: Rethinking scholarship in medical education during the era of the COVID-19 pandemic
Source: MedEdPublish (2016). 2020 May 13;9:97. [Version 1] doi: 10.15694/mep.2020.000097.1 (PMC10697454; doi:10.15694/mep.2020.000097.1)
Supplement: Peer review history

# Reviewer report: Reviewer response for version 1

**DOI:** 10.21956/mep.20079.r30901

**Published:** 2020-05-21

**Authors:** Hui Meng Er

**Affiliations:** 1 International Medical University

This review has been migrated. The reviewer awarded 5 stars out of 5 This is an excellent paper that gives new perspectives to scholarship in medical education. As health professional educators worldwide are facing enormous stress in having to adapt quickly to the changes in education delivery brought about by the COVID-19 pandemic, they are expected to continue scholarship in medical education while coping with the increased workload in teaching and healthcare delivery. A redefinition of scholarship in medical education is timely. The iterative scholarship cycle appropriately describes the approaches taken by many institutions in the implementation of online learning in a large scale within a short period of time. It is crucial that these evidences and the lessons learnt can be shared through rapid publication approaches for the benefits of various communities of educators, particularly those who are facing resource constraints. As highlighted by the authors, such research should adhere to the standard guidance on conduct of research. This will increase its acceptance among those who may initially resist this transformational change.

Reviewer Expertise:

NA

No decision status is available

# Reviewer report: Reviewer response for version 1

**DOI:** 10.21956/mep.20079.r30902

**Published:** 2020-05-21

**Authors:** Yingzi Huang

**Affiliations:** 1 The First Affiliated Hospital, Sun Yat-sen University

This review has been migrated. The reviewer awarded 5 stars out of 5 I like this informative and insightful article, which leads to the reflection of what really matters for medical education. What really matter is not research per se but to make a difference in the real world through acting on the findings of research, especially for marginalized people under the global crisis of Covid-19 pandemic. As there are always opportunities lying within crisis, I see hope through this article that medical education will change fundamentally, achieving ' 2 years' worth of digital transformation in 2 months'. Again many thanks for such an inspiring article, reminding and empowering all of us to take actions apart from coping with and fighting against Covid-19.

Reviewer Expertise:

NA

No decision status is available

# Reviewer report: Reviewer response for version 1

**DOI:** 10.21956/mep.20079.r30900

**Published:** 2020-05-15

**Authors:** Sateesh Babu Arja

**Affiliations:** 1 Avalon University School of Medicine

This review has been migrated. The reviewer awarded 4 stars out of 5 A very interesting piece of work and I thoroughly enjoyed reading this paper. This manuscript is very appropriate at an appropriate time when the whole world including medical educators are going through tough trying times. One of the most important suggestions proposed by authors is that institutions should be ready for supporting the educators and accepting the metrics of scholarship on open-access journals and social media. Authors also have explained with examples of simple and complex research problems. The iterative research cycle proposed by authors reminds me of LEAP Planning and Evaluation Cycle. However, the research cycle proposed by authors looks much simpler and evaluation and planning can be done at the same time in each cycle to inform the next cycle. Thank you.

Reviewer Expertise:

NA

No decision status is available

# Reviewer report: Reviewer response for version 1

**DOI:** 10.21956/mep.20079.r30898

**Published:** 2020-05-13

**Authors:** Subha Ramani

**Affiliations:** 1 Harvard Medical School, Brigham and Women's Hospital

This review has been migrated. The reviewer awarded 5 stars out of 5 I enjoyed reading this article. It is a timely reflection on the many types of scholarship that educators can pursue. The authors emphasize that research is not the only mode of scholarship. However, peer review and dissemination are essential to call an output scholarship. Boyer's definition of scholarship, types of scholarship should be reviewed by all teachers, educators and educationalists. Glassick's criteria for sound educational scholarship inform how to make an educational product count twice, yet be open for peer review and reflective critique. This then leads to ongoing monitoring and revising of educational initiatives.I would add that all health professions educators should read the AMEE guide on "Research Compass", which emphasizes not confining oneself to the local educational context. Applying principles from all these models and frameworks would be of great value to guide the scholarship of teaching and learning.I agree that journals and editors should keep pace with the plethora of scholarly work that can be of use to the world of education and not stick to anachronistic traditions.The point of scholarship is sharing useful educational work and products with a global community of teachers and learners as well as educational scholars and leaders.

Reviewer Expertise:

NA

No decision status is available

# Reviewer report: Reviewer response for version 1

**DOI:** 10.21956/mep.20079.r30899

**Published:** 2020-05-13

**Authors:** Tom Grimwood

**Affiliations:** 1 University of Cumbria

This review has been migrated. The reviewer awarded 5 stars out of 5 This is a very timely piece and offers a number of challenges to conventional scholarship not only in the field of medical education, but in general as well. The opportunities for reconceptualising research are important to focus on.I find the case well made for reviewing the ways in which research, scholarship and teaching interact in the light of COVID-19. I do wonder if, when developing this line of thought, the authors would consider the different audiences or interlocutors in play. For example, some of the issues raised here are long-established tensions (e.g. the lack of recognition for alternative research methods), whereas others are more localised and transient (e.g. the changes to on-line education, which will depend much on educator experience, available technology and student body). It seems to me that the interplay between the macro and micro issues will affect decision-making about teaching, and so the move to suggesting metrics for capturing this is a little too quick (for me). Likewise, a sense of whether this transformation would be a ground-up, top-down or hybrid approach might be worth a further discussion (similar to, say, Sanford Schram's work in social policy).

Reviewer Expertise:

NA

No decision status is available
